# Notch signaling in glioblastoma: a developmental drug target?

**DOI:** 10.1186/1741-7015-8-72

**Published:** 2010-11-15

**Authors:** Maria Maddalena Lino, Adrian Merlo, Jean-Louis Boulay

**Affiliations:** 1 Laboratory of Molecular Neuro-Oncology, Department of Biomedicine, University Hospital Basel, Basel, Switzerland; 2 Laboratory of Brain Tumor Biology, Department of Biomedicine, University Hospital Basel, Basel, Switzerland

## Abstract

Malignant gliomas are among the most devastating tumors for which conventional therapies have not significantly improved patient outcome. Despite advances in imaging, surgery, chemotherapy and radiotherapy, survival is still less than 2 years from diagnosis and more targeted therapies are urgently needed. Notch signaling is central to the normal and neoplastic development of the central nervous system, playing important roles in proliferation, differentiation, apoptosis and cancer stem cell regulation. Notch is also involved in the regulation response to hypoxia and angiogenesis, which are typical tumor and more specifically glioblastoma multiforme (GBM) features. Targeting Notch signaling is therefore a promising strategy for developing future therapies for the treatment of GBM. In this review we give an overview of the mechanisms of Notch signaling, its networking pathways in gliomas, and discuss its potential for designing novel therapeutic approaches.

## Introduction

Gliomas are defined as brain tumors of glial origin. Based on histology, gliomas have been classified into astrocytoma (70%), oligodendroglioma (10% to 30%), mixed oligoastrocytoma and ependymoma (<10%). Low-grade gliomas, mostly astrocytomas (World Health Organization (WHO) grade II) are progressively transforming into malignant gliomas, that is, anaplastic tumors (WHO grade III) and ultimately into glioblastoma multiforme (GBM; WHO grade IV). However, most GBM are diagnosed without any prior record of a tumor of lower grade [1,2]. GBM is a complex mixture of cell types that includes astrocyte-like and stem-like cells, characterized by rapid growth and diffuse invasiveness into adjacent brain parenchyma. Resectability depends on tumor location and only the nodular component can be surgically controlled. The infiltrative component of the tumor, however, is left to unspecific and cytotoxic chemotherapy and radiotherapy that can impede tumor progression for a limited time only. GBM patient survival is of less than 1 year [2,3]. GBM has a severe mutator phenotype that consists of large chromosomal alterations [4,5]. At the genetic level, the most frequent mutations affect genes involved in the control of cell cycle, growth, apoptosis, invasion and neovascularization [6,7]. In the past few years, it has become apparent that Notch signaling, a major player in normal development of the central nervous system, is often misregulated in GBM. In this review we will focus on the role of Notch in gliomagenesis and discuss potential therapeutic opportunities.

### Notch: genetics, biology and signaling

#### Pioneer observations on Notch in *Drosophila*

The *Notch *mutation was discovered by Thomas Morgan in 1917 in the fruit fly *Drosophila melanogaster*, with an adult phenotype consisting of 'notches' at the wing margin. Genetic analyses of *Notch *loss-of-function mutations also revealed an embryonic phenotype with an expanded population of neuroblasts at the expense of epidermis cells. These mutations provided the first clue that during neurogenesis, wild-type Notch regulates the cell fate decision by preventing ectoderm cells from differentiating into neuroblasts rather than into epidermis, and have been therefore qualified as neurogenic mutations [8]. Further identification of antineurogenic gain-of-function mutations completed the description of the allelic series of *Notch *[9,10]. Both loss-of-function and gain-of-function *Notch *mutations are dominant in *Drosophila*, where loss and gain of a single gene copy is sufficient to mimic hypomorphic and hypermorphic mutations [9-11]. Thus, the *Notch *expression level is likely to be critical to ensure the subtle balance between neuroblast and epidermal cell fate decision during *Drosophila *development.

### Cloning of *Notch *genes

**Figure 1:**
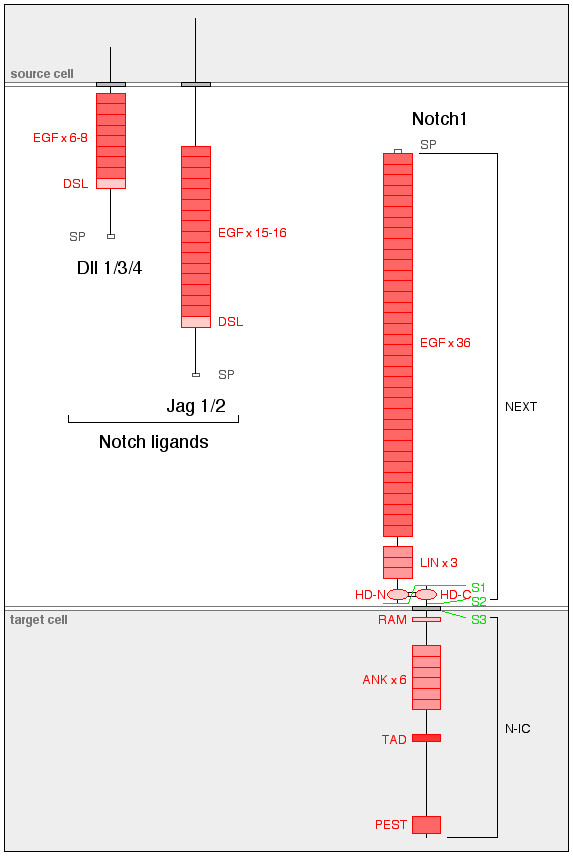
**Ligands, structure and processing of Notch1 receptor**. Left: the Delta-like ligand (Dll) and Serrate-Jagged ligand (Jag) structural subsets of Notch ligands. Right: structure of Notch1 receptor resulting from post-translational cleavage at site S1 and heterodimerization of the cleaved parts. Ligand-dependent cleavages at sites S2 and S3 generate soluble Notch extracellular truncation (NEXT) and cytosolic Notch intracellular domain (N-IC) forms, respectively. Notch1 and Notch2 are highly similar. Notch3 and Notch4 contain 34 and 29 epidermal growth factor (EGF)-like repeats, and are devoid of trans-activation domain (TAD) domains.

Cloning of the *Drosophila Notch *gene [12] revealed a type I transmembrane receptor consisting of 36 epidermal growth factor (EGF)-like tandem repeats and 3 cysteine-rich LIN-12/Notch (LIN) repeats in the extracellular domain. The extracytoplasmic juxtamembrane region forms both N-terminal and C-terminal heterodimerization domains (HD-N and HD-C, respectively). The cytoplasmic part contains an RBPJk-binding (RAM) domain, six tandem ankyrin (ANK) repeats, a transcription activation domain (TAD) and a proline/glutamate/serine/threonine-rich (PEST) sequence. Post-translational cleavage of the single Notch receptor chain at site S1 located between HD-N and HD-C domains and subsequent heterodimerization between HD-N and HD-C generates a functional receptor [13,14]. Notch1 ligands, receptor domains and processing are illustrated in Figure 1. Vertebrate genomes encode four Notch paralogs, where Notch1 and Notch2 show strong structural homology with *Drosophila *Notch. Notch3 and Notch4 are more distantly related, with 34 and 29 EGF-like repeats, are and devoid of TAD domains [15-18].

### Expression pattern in mammalian brain

In rodent late embryonic and postnatal brain, Notch1, Notch2 and Notch3 transcripts are commonly present in germinal zones, but with distinct patterns and later postembryonic expression of Notch2 [19,20]. In postnatal mouse brain, Notch2 expression persists in glial cells harboring markers of immature phenotype: high vimentin and low glial fibrillary acidic protein (GFAP) [21]. Consistent with expression in immature glial cells in the germinal zones, Notch signaling is required for preventing neuronal differentiation and promoting neural stem cell (NSC) maintenance for further commitment into glial lineage. Maintenance of the NSC population by Notch signaling prefigures a possible role of Notch signaling in the maintenance of glioma stem cell (GSC) population [22-26].

### Notch signaling mediators

**Figure 2:**
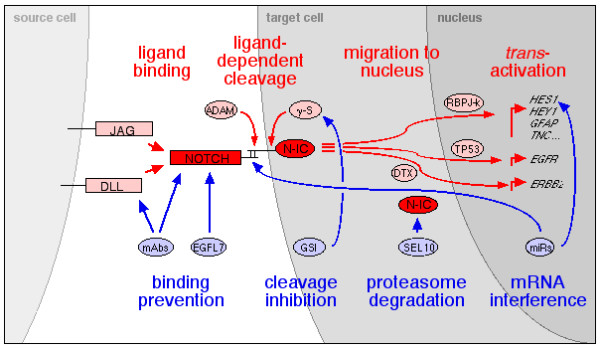
**Sequential events and control of Notch signaling**. Activating and inhibitory mechanisms are depicted in red and blue, respectively.

In mammals, Notch receptors are activated by five type I transmembrane ligands, three Delta-like (Dll1, Dll3 and Dll4) and two Serrate/Jagged (Jag1 and Jag2) receptors (Figure 1). All contain a cysteine-rich 'Delta, Serrate, Lag' (DSL) motif found in *Drosophila *respective orthologs Delta and Serrate/Jagged and in *Caenorhabditis elegans *Lag2. Numbers of EGF repeats vary between Dll and Jag ligands (6-8 and 15-16, respectively) [27] Recently, epidermal growth factor-like domain 7 (EGFL7) has been identified as a soluble antagonist of Notch signaling [28]. Ligand-dependent cleavage at site S3 within the transmembrane domain (Figure 1) of the membrane-bound receptor releases a Notch intracellular (N-IC) form, which translocates to the nucleus. There, it binds Su(H)/CSL/CBF1/RBPJk to *trans*-activate target genes such as the hairy/enhancer of Split *HES *and *HEY *families of basic helix-loop-helix transcription factors [27,29,30]. These successive events are dissected in the upper part of Figure 2. An additional ligand-dependent cleavage at extracellular site S2 (Figure 1) leads to the release of a soluble form of Notch named Notch extracellular truncation (NEXT) [31].

Further, a non-canonical RBPJk-independent and Deltex-dependent alternative pathway has been described in humans and in *Drosophila *[32,33]. Together with this observation, in T helper (Th) cells, Jagged induces Th2 cell differentiation by triggering the RBPJk-dependent canonical pathway, while Delta-like instructs Th1 commitment through a RBPJk-independent alternative pathway, presumably Deltex-dependent [34].

Physical interactions between Notch target gene products HES1 and HEY1 with Stat3 point to crosstalks between Notch and Stat3-activating pathways such as Gp130/Jak2/stat3 and Sonic hedgehog (Shh) [25,35,36]. In parallel, Shh is also capable of stimulating *HES1 *transcription [37]. In addition, β-catenin has been shown to interact with Notch and RBPJk to induce *HES1 *transcription, indicating also crosstalk between Wnt and Notch pathways [26]. Levels of crosstalks between Notch and these pathways are discussed in Hansson *et al*. [38].

### Notch germline mutations, human diseases and knockout models

In humans, *Notch *mutations have been associated with dominant developmental disorders and diseases that include brain/neurological, cardiovascular and/or kidney defects. *Notch1 *in aortic valve disease [39]; *Notch2 *in Alagille syndrome [40]; *Notch3 *in Cadasil syndrome [41] and possibly *Notch4 *in schizophrenia [42]. In mice, global Knockouts of *Notch1 *or *Notch2 *are embryonic and perinatal lethals with vascular and kidney defects [43,44]. Surprisingly, *Notch3 *and *Notch4 *null mice showed normal development, viability and fertility. Although *Notch1/Notch4 *double mutants had more severe defects in angiogenic vascular remodeling, there is no evidence of a genetic interaction between *Notch1 *and *Notch3 *[45,46]. Hemizygosity of *Dll4 *as well as *Jag1 *and *Rbpjk *Knockouts consistently result in embryonic death due to vascular defects [47].

In fact, occurrence of disorders with embryonic or perinatal lethality are likely to mask the involvement of Notch signaling in later developmental or biological events. This includes GBM progression, onset of which occurs in human adults at the mean age of 62 [2]. Nevertheless, the fact that inactivation of Notch signaling results in constant defects in angiogenesis shows its role in vascular morphogenesis and remodeling during embryonic development, and reveals a possible involvement of Notch signaling in tumor neovasculature.

### Notch and cancer

Notch signaling plays a pivotal role in the regulation of many fundamental cellular processes such as proliferation, stem cell maintenance, differentiation during embryonic and adult development and homeostasis of adult self-renewing organs [27,48]. Therefore, it is easy to see how perturbation of Notch signaling may often lead to tumorigenesis.

### Notch and malignancy

The first evidence for a role of Notch in tumorigenesis came from the finding that the acute T cell lymphoblastic leukemia translocation (T-ALL) t(7;9)(q34;q34.3) breakpoint generated the fusion of the gene for the ß chain of the T cell receptor at 7q34 and the *TAN1/NOTCH1 *gene at 9q34.3, giving rise to a constitutively active N-IC-like domain [49]. More generally, acquired gain-of-function Notch mutations in T-ALL cluster at HD domains to form constitutively active receptors, and at the PEST domain to stabilize active N-IC [50]. Similarly, a fraction of B cell lymphomas harbor mutations in the PEST domain of Notch2 [51]. Further, Notch proteins have been shown to be involved in tumors of various origins. However, oncogenic or tumor suppressive activities of Notch depends on the cellular context [52] or might be a matter of Notch expression level, as observed in neural stem cells [53]. In other neoplasms, such as non-small cell lung cancer and skin cancer, Notch has a tumor suppressor function [6,7].

As discussed in the next section, Notch signaling is one of the major pathways involved in GBM development. Notch signaling has been shown to maintain proliferation of normal neural precursors and has been defined as a survival marker in gliomas [23-25,54]. Its oncogenic function in gliomas is based on cell proliferation and invasion [55,56]. Data on other tumor cell types showing the role of Notch in epithelial-to-mesenchymal transition (EMT) in response to hypoxia may be extrapolated to GBM [57,58].

### Notch and GBM

GBM is the most aggressive central nervous system (CNS) tumor, with the poorest clinical prognosis. This tumor consists of cells that are astrocyte-like but with a complex genetic make-up and expression patterns in which the presence of stem-like cells has been proven [2,3,59]. Notch2 has been suggested to drive embryonic brain tumor growth, whereas Notch3 has been implicated in choroid plexus tumors [23,60]. In GBM and in medulloblastoma, the frequency and the intensity of Notch2 expression is higher than that of Notch1 [55,61]. As a consequence of local genomic amplifications at the *Notch2 *locus in both brain tumor types, this may also be linked to the later persistence of *Notch2 *expression in postnatal mouse brain [21]. In fact, in medulloblastoma, Notch2 is preferentially expressed in proliferating progenitors, while Notch1 in postmitotic differentiated cells [61]. Interestingly, Purow *et al*. showed that Notch1 regulates transcription of the epidermal growth factor receptor gene *EGFR*, known to be overexpressed or amplified in GBM, through TP53 [62]. Consistently, transcription of Notch signaling mediator genes are significantly overexpressed in the molecular subset of GBM with *EGFR *amplification [63]. This new link places Notch signaling as an activator of the major GBM pathway and further clarifies the implication of Notch signaling in cancer and development.

In contrast, a minor GBM subset with local haploidy at 1p12 has been identified and has been associated with better patient prognosis. Reminiscent of the better outcome of oligodendroglioma patients harboring 1p/19q loss, the minimal area of loss in GBM and the detection of homozygous deletions in oligodendroglioma converge to the *Notch2 *gene [54]. This provided an initial clue that subsets of gliomas (even with distinct histologies) with impaired Notch signaling result in slower progression. A single loss-of-function mutation in the RAM domain of Notch2 has been identified in the glioma line Hs683. This mutation has been further shown to impair Notch-mediated *trans*-activation, and subsequently tenascin-C (TNC)-mediated invasion, as detailed below [55].

### Effect on proliferation and apoptosis by Notch modulation in GBM

Genomic amplification of *EGFR *is the most frequent genetic alteration occurring in GBM, a fraction of which undergoes a further deletion that generates the constitutively active *vIII *variant [64,65]. Consistently, EGF is the major proliferation pathway in GBM, mediated by activation of the RAS-RAF-MEK-ERK and the PI3K-AKT-mTOR cascades [66]. Interestingly, mTOR has recently been shown to activate Notch signaling in lung and kidney tumor cells through induction of the Stat3/p63/Jagged signaling cascade [67]. If true in GBM, this potentially creates a positive feedback loop between Notch and EGF signaling. The most frequent GBM subset consists of the association of *EGFR *amplification together with homozygous deletions at the *CDKN2A *(cyclin dependent kinase) locus, and mutually exclusive of *TP53 *mutations [5]. Since Notch has been shown to activate expression of EGFR via TP53 [62], Notch is expected to stimulate the main GBM proliferation pathway. Of note, the gene for the EGFR-related ERBB2 is also *trans*-activated by Notch, but in a DTX1-dependent manner [68].

Notch pathway inhibition by γ-secretase inhibitors (GSIs) reduced GSC proliferation and increased apoptosis associated with decreased AKT and Stat3 phosphorylation [69]. Conversely, expression of an active form of Notch2 increased tumor growth and *in vivo *delivery of GSI consistently blocked tumor growth, and significantly prolonged survival [69]. Taken together, these results open inhibition of Notch signaling as a promising strategy to control GSC growth.

### EMT and invasion

EMT represents the transition through which a benign tumor becomes malignant. Underlying molecular changes lead to decreased cell adhesion and acquisition of tumor invasiveness [70]. Together with transforming growth factor (TGF)β, the Jag1-Notch pathway activates *HEY1 *to trigger EMT of epithelial cells of human, murine and canine origins [71]. The Jag1-Notch-Snai2 cascade has also been showed to induce EMT in human breast tumor cell [57]. Unlike these tumors in which invasion results into remote metastases, GBM invade adjacent brain tissue. Further studies may validate the same molecular mechanism in GBM local invasion.

TNC, highly expressed in invasive GBM, is an extracellular matrix glycoprotein [72] that induces proliferation and migration of neuronal precursors in embryonic and postnatal mouse brain neurogenic zones [73], and ensures neuronal regeneration in injured adult brain [74]. TNC levels increase during progression of GBM, such that it can be used as a prognostic marker for GBM patient survival [75]. The molecular mechanism through which Notch signaling induces TNC-dependant glioma cell motility is based on the *trans*-activation of the *TNC *promoter by Notch-RBPJk [55]. It is noteworthy that a parallel study in childhood ependymomas has shown an association between tumor recurrence and frequent amplification of the 9qter, precisely at the location of both *NOTCH1 *and *TNC *genes [76].

### Glioma differentiation and stem cells

In recent years, it had been suggested that within a tumor, only a subset of cells called cancer stem cells (CSCs), are endowed with tumorigenic capacity [77-79]. These cells are self-renewing and multipotent with tumor initiating potential. The alternative hypothesis that transformation and dedifferentiation of more mature brain cells contributes to tumorigenesis may be a parallel pathway towards tumorigenesis [80]. Notch signaling is crucial for the *in vivo *maintenance of self-renewing stem cells in varieties of lineages such as neural, but also hematopoietic or mammary [81-83]. Similar to Notch-RBPJk, Stat3 *trans*-activates *GFAP *and induces normal neural precursor cells to differentiate into astrocytes in association with HES1, HES5 or HEY1. At the molecular level, HES1 physically interacts with Jak2 and Stat3 to induce Stat3 phosphorylation and relevant activation [25,35,84]. Conversely, the association between Notch, RBPJk and β-catenin maintains the precursor cell status [26].

Notch2 and HES1 have been shown to maintain the granule neuron progenitor cell population and inhibit further neuronal differentiation [23]. Reciprocally, brain-derived EGFL7 regulates Notch-dependent proliferation and differentiation of NSC of the subventricular zone into neurons and oligodendrocytes [28]. Further, neuron-induced Jag1-Notch1 signaling upregulates expression of the radial glia marker brain lipid binding protein (BLBP) [68]. In addition, the gene for Nestin, a marker for neural precursors, is *trans*-activated by Notch signaling [85]. Consistently, in ependymoma cells, the fraction of CD133-positive cells shows significant coexpression of Notch2 and HES1 [72]. This supports a function of Notch signaling in maintaining the GSC population within a tumor. However, Notch signaling has also been shown to *trans*-activate the gene for GFAP [86] and to drive the differentiation of glial progenitor cells into astrocytes therefore preventing differentiation into oligodendrocytes [87]. This is consistent with the observation that Notch1 and Notch2 are present at high levels in GBM and astrocytoma [55]. These distinct activities suggest multiple roles for Notch signaling in the course of gliomagenesis, in particular in GBM and astrocytoma development, that remain to be dissected. In oligodendrogliomas, *Notch2 *is frequently deleted and the corresponding protein is not detectable [54,55].

MicroRNAs (miRNAs) are short single-stranded RNAs that negatively regulate gene activity by targeting mRNAs for cleavage or translational repression [88]. The miRNA miR-199b-5p has been recently identified as a regulator of the Notch pathway by targeting the transcription factor HES1. Its overexpression blocks expression of several CSC genes and decreases the medulloblastoma stem-cell-like (CD133+) subpopulation of cells [89]. Similarly, treatment of ependymoma neurospheres with GSI-IX results in decrease sphere number, size, proliferation and induced cell-surface adhesion [76]. GSI have been proved to significantly reduce radioresistance of glioma stem cells through inhibition of Notch [90].

GSCs represent a critical therapeutic target to control glioma growth and progression. However, the molecular mechanisms that regulate the stem cell pool are poorly understood. The vascular, perinecrotic and hypoxic niches of the tumor constitute a microenvironment that contributes to the regulation of CSC. Hypoxia plays a key role in the regulation of the CSC phenotype through hypoxia inducible factor (HIF)-2α and subsequent induction of specific CSC signature genes, including mastermind-like protein 3 (Notch pathway), nuclear factor of activated T cells 2 (calcineurin pathway) and aspartate β-hydroxylase domain-containing protein 2 [91].

MicroRNA-34a (miR-34a) is a transcriptional target of TP53 that is downregulated in GBM and even more in GBM carrying a TP53 mutation, as compared to normal brain. Transient expression of miR-34a in GBM cells strongly inhibited glioma xenograft growth *in vivo *by targeting *c-Met *and *Notch *[92].

### Hypoxia and angiogenesis

GBM possesses a chaotic tumor structure consisting of accumulating tumors cells, abnormal vessel and necrotic debris. The increasing tumor mass leads to pressure gradient leading to capillary and venous collapse [66]. The new formed vessels are structurally and functionally abnormal, and leaky, giving rise to edema, high interstitial fluid pressure and, consecutively, low oxygen tension [93]. In contrast to high O_2 _tension, which degrades HIF-1α (hypoxia inducible factor) and promotes differentiation or apoptosis of NSCs, lower O_2 _tension *HIF-1α *facilitates signal transduction pathways that promote self-renewal [94]. This hostile microenvironment selects for a more malignant phenotype by clonal outgrowth of hypoxia-resistant tumor cells.

Genetic models have shown the role of Notch in normal arteriogenesis and neoangiogenesis [95]. Its influence is also crucial for neovascularization in cancer contributing to the aggressive clinical behavior of tumors expressing high levels of Notch ligands [96,97]. Recently, Notch1 has been shown to upregulate *HIF-1α *expression in breast cancer. In turn, HIF-1α binds and stabilizes activated Notch to enhance Notch signaling [73,97,98]. Thus, O_2_, HIF and Notch regulation may play together a crucial role also to the normal architecture and dynamics of NSC regulation.

### Manipulating the Notch network in brain tumors for therapeutic benefit

Manipulating the Notch pathway would directly and indirectly influence all the downstream and collateral pathways that interact with the complex Notch family signaling.

### Notch signaling target genes

As described above, Notch signaling *trans*-activates and upregulates genes expressed in gliomas. Canonical Notch/RBPJk-dependence has been shown for *GFAP *[86], *HES1 *[87], *HEY1 *[71], *BLBP *[99], *NESTIN *[85], *TNC *[55] genes while non-canonical DTX1-dependent *trans*-activation has been shown for *ERBB2 *[68]. Finally, Notch1 induces TP53-dependent EGFR expression [62]. However, the molecular genetic mechanism of this latter cascade remains to be elucidated. Thus, the oncogenic role attributed to Notch signaling is acting through a complex array of effector genes.

### Notch signaling regulation

Interestingly, *TNC *and *HEY1 *are commonly *trans*-activated by Notch and TGFβ signaling [71,100,101]. In fact, Notch is also upregulated by sex determining region Y box 2 (SOX2), SOX2 by SOX4, and SOX4 by TGFβ [93,102,103], while *JAG1 *is upregulated by TGFβ and Wnt [104,105]. This shows the different levels through which Notch, TGFβ and Wnt pathways act in a concerted and synergistic manner.

### Clinical studies using Notch inhibitors

Notch signaling has emerged as a specific therapeutic target for T-ALL [50] and colon cancer [106], as well as a potential target against tumor angiogenesis [105,107,108]. Blocking of Notch pathway induces apoptosis and depletes cancer stem cells in medulloblastoma [109]. A phase I study of GSI MK0752 for adult and pediatric patients with relapsed or refractory acute t-cell lymphoblastic leukemia and lymphoma is ongoing (NCT00100152). More recently, a phase I study of MK0752 was initiated in patients with metastatic or locally advanced breast cancer and other solid tumors (NCT00106145). A new clinical trial has just started for treating patients with recurrent or progressive GBM using GSI RO4929097 (NCT01122901) http://clinicaltrials.gov/ct2/show/NCT01122901?term=notch&rank=14.

The difficulty of distinguishing Notch1 and Notch2 specific activities from one another in GBM modulating proliferation, angiogenesis, invasion and cancer stem cell maintenance suggests by default that both are mostly redundant. Indeed, the pharmacological approaches using GSI do not discriminate for a specific Notch receptor and cause gastrointestinal toxicity as side effect [106]. However, based on the specific role of Dll4-Notch1 in neovascularization, anti-Dll4, and similarly anti-Notch1 and anti-Notch2 antibodies have been proposed as sharper therapeutic agents devoid of side effects against various tumor types in mouse xenograft models [107,110]. The use of mouse glioma models put into specific Notch mutant backgrounds may help to solve this issue. Further downstream, the role of non-canonical Notch pathway has not been clarified yet and more in-depth studies will be needed to define the effect on Notch canonical pathway.

### Concluding remarks and discussion

GBM is the most prevalent and the most aggressive brain tumor against which conventional therapies, that is, radiotherapy, chemotherapy and surgery have led until now to only transient clinical response followed by tumor recurrence, with no significant improvement of patient survival. Notch signaling has recently been identified to cumulate oncogenic activities in GBM proliferation, apoptosis inhibition and invasion. Additional functions in maintaining non-neoplastic neural stem cells and in neovascularization and in EMT switch of other malignancies remain to be demonstrated in GBM. With the development of novel therapies interfering with identified cancer pathways, Notch pathway therefore holds a promise of being a particularly appropriate target to fight against GBM.

Prerequisites need to be fulfilled by a compound potentially designed for targeted GBM therapy. The drug needs to be harmless for healthy cells and to be able to pass the blood brain barrier to penetrate the tumor. Further, as previously shown *in vitro *[111,112], combinations of compounds that target non-redundant GBM pathways or with cytotoxic agents may synergize to induce GBM cell death. Such combinations that would include Notch signaling inhibitors are hoped will provide promising therapies to substantially improve patient outcomes.

## Competing interests

The authors declare that they have no competing interests.

## Authors' contributions

ML and JL Boulay assisted in the review conceptualization, design and writing of the manuscript. AM assisted with the review and in writing the manuscript.

## Pre-publication history

The pre-publication history for this paper can be accessed here:

http://www.biomedcentral.com/1741-7015/8/72/prepub
